# RNA-seq assembly and analysis of *Garcinia mangostana* transcriptome during seed germination

**DOI:** 10.1016/j.dib.2017.07.064

**Published:** 2017-08-02

**Authors:** Nur Diyana Kamal Azlan, Mohd Noor Mat Isa, Zamri Zainal

**Affiliations:** a Institute of Systems Biology, Universiti Kebangsaan Malaysia, UKM, 43600 Bangi, Selangor, Malaysia; b Malaysia Genome Institute, Jalan Bangi, 43000 Kajang, Selangor, Malaysia; c School of Bioscience and Biotechnology, Faculty of Science and Technology, Universiti Kebangsaan Malaysia, UKM, 43600 Bangi, Selangor, Malaysia

**Keywords:** Transcriptome, *Garcinia mangostana*, Seeds, Germination

## Abstract

*Garcinia mangostana* is a tropical fruit plant rich in antioxidant and bears recalcitrant seeds. The extent of water loss and low temperature tolerable by recalcitrant seed varies from regular orthodox seeds. Present study generates transcriptome resources for *G. mangostana* to postulate potential transcriptome differences between recalcitrant and orthodox seeds during seed germination process. Raw reads of pooled samples used for the assembly have been deposited in genbank accession SRR5412332.

**Specifications Table**

**Table:** 

Subject area	*Biology*
More specific subject area	*Molecular biology*
Type of data	*Transcriptome data*
How data was acquired	*Paired-end sequencing was performed on a HiSeq. 2000. De-novo transcriptome assembly was carried out using Oases v0.2.08 (*http://www.ebi.ac.uk/~/zerbino/oases/*). The appropriate k-mer and coverage cut-off value was determined by a perl script (VelvetOptimiser-2.2.0.pl).*
Data format	*Raw sequences*
Experimental factors	*Post-imbibed seeds vs. control seeds. Each sampel contain three biological replicates ane each replicates are pool of ten seeds.*
Experimental features	*Total RNA was extracted from post-imbibed seeds of Garcinia mangostana at day 0, day 3, day 5 and day 7. Control seeds were harvested from the fruits*.
Data source location	*Seeds of Garcinia mangostana was sown in the glass house at UKM Bangi, Selangor, Malaysia (2°55′14.5′′N 101°47′01.4′′E)*
Data accessibility	*Deposited in SRA database with accession number SRR5412332 (*https://www.ncbi.nlm.nih.gov/sra/SRR5412332/*)*

**Value of the data**

• *Garcinia mangostana* is a non-model tropical fruits plant which produces recalcitrant seeds.
• Current transcriptome improve transcriptomic database of *Garcinia mangostana*.
• Improved transcripts repositories with increased KEGG pathways coverage provide extensive genetic resources to integrate research of orthodox and recalcitrant seeds.
• This data will add transcriptomic resources for further study of molecular regulation of seed germination in recalcitrant seeds.

## 1 Data

To profile the seed transcriptome of *Garcinia mangostana*, RNA-seq short reads were generated from cDNA libraries prepared from the total RNA extracted from germinating seeds. The short reads were filtered, processed, assembled and analysed as described below. The raw data of pooled samples used for the assembly project have been deposited at genbank under the accession SRR5412332. Individual samples of day 0, day 3 day 5 and day 7 were each deposited in genbank under the accession SRR5412331, SRR5412330, SRR5412329 and SRR5412328 accordingly.

## 2 Experimental design, materials and methods

### 2.1 Plants materials

Sampling of *G. mangostana* fruits were done from the experimental plot (2°55′09.0″N 101°47′04.8″E) at Universiti Kebangsaan Malaysia, Bangi. The seeds were harvested from the fruits with flesh peeled seeds rinsed with distilled water. Seed were divided according to the experimental design. Control seeds were frozen in liquid nitrogen and stored under −80 °C after harvested from the fruits. Seeds subjected for germination were planted in soil, watered and collected accordingly at day 0, day 3, day 5 and day 7 post-imbibition. Collected seeds were frozen in liquid nitrogen and stored under −80 °C.

### 2.2 Total RNA extraction, quality control, library preparation and RNA-seq

Total RNAs were extracted accordingly based on protocols reported by Lopez-Gomez [1]. A cDNA library was constructed with the TruSeq RNA Sample Prep kit (Illumina, USA) using minimum of 4000 ng of total RNA that was prepared from *G. mangostana* seed sample. The total RNA from day 0, day 3, day 5 and day 7 were pooled, tagged differently to distinguish the different samples and sequenced on a HiSeq. 2000 (Illumina, USA) with paired-end 90 bp read lengths.

### 2.3 Transcriptome de novo assembly, annotation and classification

The reads were trimmed and filtered with FASTX-Toolkit to eliminate the low quality reads. Reads were considered as high quality if more than 70% of the bases had phred values of more than Q20 and were kept for assembly. Reads shorter than 30 bp after trimming were discarded from further analysis. Perl script *select_paired.pl* was used to separate paired ends from singletons sequences. Sequences with pairs were combined in alternating sequence using *shuffleSequences.pl*. Reads produced in this study have been deposited in the NCBI Sequence Read Archive (SRA) (accession number SRP075857).

Final assembly was carried out using Oases v0.2.08 (http://www.ebi.ac.uk/~/zerbino/oases/) [2]. The appropriate k-mer and coverage cut-off value used in the assembly was determined by a perl script (VelvetOptimiser-2.2.0.pl). K-mer size range from 75 to 89 were tested for the best N50 value while the coverage cut-off was automatically determined by the script. All other parameters were on default settings. Transcripts with a minimum length of 200 bp obtained from final assembly were used for annotation and further analysis. Statistic of the assembly is showed in Table 1.

**Table 1:** Sequencing and RNA-seq statistics of *Garcinia mangostana* Transcriptome During Seed Germination.

Attributes	Value
**Pre assembly**	
Total raw reads	40,870,131
Total processed reads	40,870,131
**Post assembly**	
Total reads used for assembly	23,439,437
Percentage of reads used for assembly	57%
Total unigenes generated	27,080
Average length of generated unigenes	488
